# Protein–ligand interactions investigated by thermal shift assays (TSA) and dual polarization interferometry (DPI)

**DOI:** 10.1107/S1399004714016617

**Published:** 2015-01-01

**Authors:** Morten K. Grøftehauge, Nelly R. Hajizadeh, Marcus J. Swann, Ehmke Pohl

**Affiliations:** a Chemistry Department, Durham University, South Road, Durham DH1 3LE, England; b Farfield, Biolin Scientific, 62 Wellington Road South, Stockport, Cheshire SK1 3SU, England; c Chemistry Department and School of Biological and Biomedical Sciences, Durham University, South Road, Durham DH1 3LE, England

**Keywords:** thermal shift assays, dual polarization interferometry, protein–ligand interactions

## Abstract

Over the last decades, a wide range of biophysical techniques investigating protein–ligand interactions have become indispensable tools to complement high-resolution crystal structure determinations. Current approaches in solution range from high-throughput-capable methods such as thermal shift assays (TSA) to highly accurate techniques including microscale thermophoresis (MST) and isothermal titration calorimetry (ITC) that can provide a full thermodynamic description of binding events. Surface-based methods such as surface plasmon resonance (SPR) and dual polarization interferometry (DPI) allow real-time measurements and can provide kinetic parameters as well as binding constants. DPI provides additional spatial information about the binding event. Here, an account is presented of new developments and recent applications of TSA and DPI connected to crystallography.

## Abstract 2

The biophysical characterization of protein–ligand interactions in solution using techniques such as thermal shift assay, or on surfaces using, for example, dual polarization interferometry, plays an increasingly important role in complementing crystal structure determinations.

## 1. Introduction   

The biophysical characterization of protein–ligand relations ranging from protein–ion and protein–drug interactions to protein–protein and protein–nucleic acid interactions plays a key role in structural biology. Methods routinely used to screen compound libraries span from highly specialized techniques requiring significant instrumentation and expertise such as NMR (Sillerud & Larson, 2012 ▶) and mass spectrometry (Hofstadler & Sannes-Lowery, 2006 ▶) to simpler methods including thermal shift assays (TSA) that determine a shift in melting temperature typically measured by changes in light scattering or by fluorescence techniques. Fluorescence-based techniques are often called Thermofluor assays or differential scanning fluorimetry (DSF; Pantoliano *et al.*, 2001 ▶; Semisotnov *et al.*, 1991 ▶; Vedadi *et al.*, 2006 ▶). TSA can easily be performed in most laboratories and are now routinely used in drug discovery to identify new ligands in a high-throughput mode (Winter *et al.*, 2012 ▶). In addition, TSA have been used by a number of groups to optimize crystallization conditions (Ericsson *et al.*, 2006 ▶; Geders *et al.*, 2012 ▶; Nettleship *et al.*, 2008 ▶; Reinhard *et al.*, 2013 ▶; Vedadi *et al.*, 2006 ▶). A wide range of more sophisticated biophysical techniques can then be used to further characterize biomolecular interactions. Surface plasmon resonance (SPR) biosensor techniques available from various companies are used to determine kinetic parameters as well as the binding constants (typically in the range from subnanomolar to low millimolar) and stoichiometries of biomolecular interactions. SPR measurements are normally performed with intermediate throughput and hence are rarely applicable to chemical library screening. Applications and recent advances of SPR, including its important role in fragment-based drug-discovery projects, have been reviewed in a number of excellent recent articles (Homola, 2008 ▶; Patching, 2014 ▶; Rich & Myszka, 2000 ▶). Isothermal titration calorimetry (ITC) is often considered to be the most accurate method to determine not only binding constants (in a typical range from subnanomolar to submillimolar) but also thermodynamic parameters (Freyer & Lewis, 2008 ▶; Jelesarov & Bosshard, 1999 ▶; Leavitt & Freire, 2001 ▶). Being able to dissect the enthalpic and entropic contributions of biomolecular interactions also allows the study of dynamic changes in binding events (Rodgers *et al.*, 2013 ▶; Tsvetkov *et al.*, 2013 ▶; Tzeng & Kalodimos, 2009 ▶). The high level of accuracy, however, requires the careful preparation of all buffers and milligram amounts of at least one of the components at mg ml^−1^ concentrations. ITC measurements therefore represent a highly accurate but low-throughput method. More recently, microscale thermophoresis (MST) has emerged as a new and very promising method. MST is based on measuring the motion of molecules in a localized temperature gradient created by a highly focused infrared laser. This technique offers fast determination of binding constants using comparably small amounts of sample (Jerabek-Willemsen *et al.*, 2011 ▶; Seidel *et al.*, 2013 ▶). The method, however, requires specialized instrumentation at significant cost. Another relatively recent method to investigate biomolecular interactions is dual polarization interferometry (DPI), which is based on the physisorption of molecules onto the surface of a biochip. Binding events on the chip are tracked by monitoring specific interference patterns that are used to directly calculate the refractive index and the layer thickness (Cross *et al.*, 2003 ▶).

In this article, we first present a short review of two techniques, TSA and DPI, that can be considered to be at opposite ends of the spectrum of biophysical methods. Applications and new developments will then be presented, including new screens for crystallization that were designed to deconvolute the effects of individual ions, pH and types of buffers. In addition, analysis software programmed in Python is described that aids the analysis of TSA experiments. Finally, examples of dual polarization interferometry (DPI), which offers the potential to directly link thermodynamic parameters to spatial information, are shown.

## 2. Methods review   

### 2.1. Overview of thermal shift assays (TSA)   

**Figure 1:**
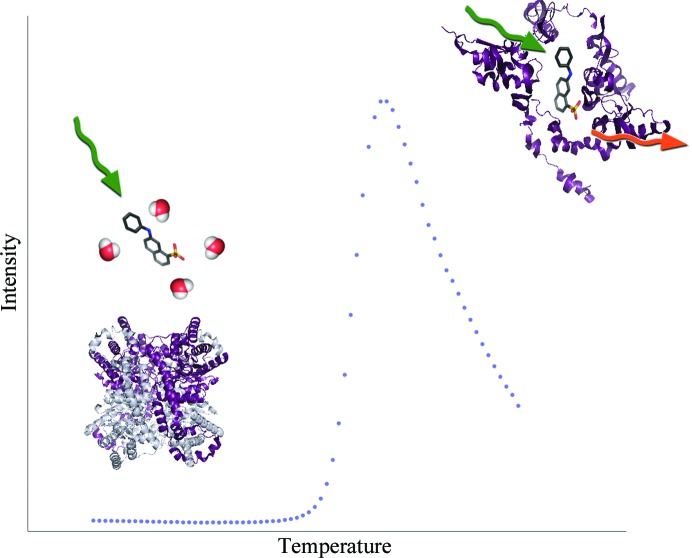
Schematic representation of a thermal shift assay showing the melting curve (blue dots) of glucose isomerase (tetramer in ribbon representation) and a single 1-anilino-8-naphthalene-sulfonate (ANS) molecule (ball-and-stick representation).

The simplest and most commonly used method for TSA is the Thermofluor assay, in which a compound with a low fluorescence signal in a polar environment (such as in aqueous solution) but with high fluorescence in a nonpolar environment is added to a protein solution (Pantoliano *et al.*, 2001 ▶). The fluorescence of the solution is monitored while the solution is heated. When the protein chain begins to unfold, the hydrophobic core becomes exposed and the signal increases until all protein molecules are completely denatured. Thus, the temperature of hydrophobic exposure at which half of the protein population is unfolded, *T*_h_, is determined (Fig. 1 ▶). A specialized variation of fluorescent TSA takes advantage of nonfluorescent compounds, such as CPM [7-diethylamino-3-(4′-maleimidylphenyl)-4-methylcoumarin], that react specifically with the side chain of free cysteine residues to form fluorescent adducts, thus revealing the temperature at which buried cysteines become solvent-accessible. This technique requires excitation at approximately 384 nm and neutral pH. Other thermal shift assays can be performed with high-throughput light scattering (Senisterra *et al.*, 2006 ▶) or by determining the temperature at which the protein is no longer able to bind to high-affinity radiolabelled ligands (Tate, 2012 ▶). A Thermofluor assay with a SYPRO dye can be performed in standard quantitative PCR instruments (Lo *et al.*, 2004 ▶). Although the assay works with most soluble protein samples under most conditions, some samples do not give a clear signal owing to denaturation to fibrils, high background caused by fluorophore binding to the protein in its native state or an insufficiently hydrophobic core. In general, increasing the thermal stability of a protein has many applications. Often, an additive that increases the thermal stability of a protein also decreases its dynamism and heterogeneity, making it more suitable for crystallography. In addition, a higher melting point of a protein also translates into a greater stability at lower temperatures, which can result in higher protein purification yields (Krintel *et al.*, 2014 ▶) and an increased probability of crystallization. Here, we have used the Thermofluor assay with SYPRO Orange. The standard protein glucose isomerase is used here as a test system to demonstrate new standard screens and new analysis tools for TSA measurements.

### 2.2. Overview of dual polarization interferometry (DPI)   

DPI shares many similarities with SPR since it is based on immobilizing one component on the surface of a biosensor. Binding events on the surface are then measured by tracking the interference pattern from two waveguides: one with the biomolecules on the surface and a second buried reference waveguide. By alternating between two orthogonal polarizations, two independent measurements of the same surface are recorded and this enables the calculation of both the refractive index and the thickness of the layer on the biosensor. These raw data allow the determination of binding constants and in addition provide real-time information concerning conformational changes upon binding (Swann *et al.*, 2004 ▶). Immobilization of a component can be achieved using different chips, ranging from unmodified surfaces to amine and thiol chips that allow binding by chemical bond formation, C18 chips optimized for binding by hydrophobic contacts and His-tags capture chips that are well suited to the His-tagged proteins frequently used in purification. Further details of the available chips can be found at http://www.farfield-group.com. DPI has been successfully used in applications ranging from biomolecular interactions to lipid structures (Hirst *et al.*, 2011 ▶) and the study of protein crystallization (Boudjemline *et al.*, 2011 ▶). Samples are first injected into a running buffer flow, which is passed over the waveguide sensor chip. First the sensor chip and running buffer refractive index are calibrated, followed by immobilization of the protein of interest onto the first of the two measurement channels, with the second channel acting as a control. Ligands can then be injected over the two surfaces. Immobilization can utilize a variety of methods from physisorption (Zwang *et al.*, 2012 ▶) and chemical coupling (Karim *et al.*, 2007 ▶) to specific binding (Coan *et al.*, 2012 ▶). In the latter case, for example, a tag such as biotin can be employed to couple the protein to a streptavidin-coated sensor chip, in which case the second control channel would usually be just streptavidin-coated. The measurements provide information on the values of, and changes in, the thickness of the layer, the refractive index/density and the mass of the protein layer. It is important to note that these values will depend on the orientation of the protein on the surface. The protein will usually have a net orientation, even if just physisorbed, but the orientation can be controlled using a site-specific tag (Coan *et al.*, 2012 ▶).

## 3. Materials and methods   

### 3.1. Thermal shift assays (TSA)   

#### 3.1.1. TSA screens   

All compounds for the two screens of 96 conditions each were purchased from Sigma–Aldrich or Fisher Scientific and were dissolved in Milli-­Q water and filtered through 0.22 µm filters. The pH screen was designed to deconvolute the effects of the pH and the buffer molecule on protein stability. The salt screen was designed to measure the effect of common salts and to deconvolute the respective effects of anionic and cationic species. The composition of the screens is given in Tables 1 ▶ and 2 ▶. The screens were designed with twice the concentration of the desired final concentration in the assay to allow assays to be set up using a 1:1 ratio of screen and protein plus dye solution. Universal buffers made with a mixture of buffer components that allow the effective control of pH over a wide range with the same buffer components (Newman, 2004 ▶) were prepared by making acidic stock solutions for each buffer with each of the three components at just above 200 m*M*. Stocks were then adjusted to pH 4 and pH 10 with NaOH, diluted to 200 m*M* and mixed in 11:0, 10:1, 9:2, …, 0:11 ratios to create a pH range with each conjugate acid–base pair at 200 m*M* to give a total of 600 m*M*. The pH was measured for each buffer at each ratio in 5° increments from 25 to 80°C to determine the Δp*K*_a_ per degree. Buffer *A* consisted of succinic acid, NaH_2_PO_4_ and glycine, buffer *B* consisted of citric acid, HEPES and CHES, and buffer *C* consisted of malonic acid, imidazole and boric acid (Newman, 2004 ▶). Equal ratios of buffer molecules were selected to simplify the deconvolution of the influence of individual species, despite resulting in a less equal spacing between pH values.

#### 3.1.2. TSA measurements   

Glucose isomerase from *Streptomyces rubiginosus* (purchased from Hampton Research; Carrell *et al.*, 1989 ▶) was buffer-exchanged with 30-­fold dilution in a spin concentrator (Sartorius Vivaspin 15, 30 kDa molecular-weight cutoff), first three times into 20 m*M* HEPES–NaOH pH 7.5, 5 m*M* EDTA–NaOH pH 7.5 and then two times into 20 m*M* HEPES–NaOH pH 7.5. The protein solution was then diluted to 1 mg ml^−1^ and aliquoted in four Eppendorf vials with 1 ml in each. 4 µl 5000× SYPRO Orange in DMSO was added to each vial and mixed by stirring with the pipette tip to give a concentration of 20 m*M* HEPES–NaOH pH 7.5, 20× SYPRO Orange, 0.4% DMSO. 2 µl 2.5 *M* MgCl_2_ were added to two of the vials to give a concentration of 5 m*M* MgCl_2_. Each were pipetted into a standard 96-well PCR plate (Starlabs Semi-Skirted FAST) with 10 µl in each well. 10 µl of salt and pH screens were pipetted into the 96-well plates with an Innovadyne Screenmaker 96+8 to give final conditions of 20 µl in each well. Both screens were repeated with MgCl_2_. Fluorescence data were collected on an Applied Biosystems 7500 FAST Real­Time PCR System with an excitation range of 510–530 nm. The fluorescence emission signal at 567–596 nm was used for data analysis. The temperature was held for 1 min per degree from 24 to 95°C.

#### 3.1.3. TSA data analysis   

**Figure 2:**
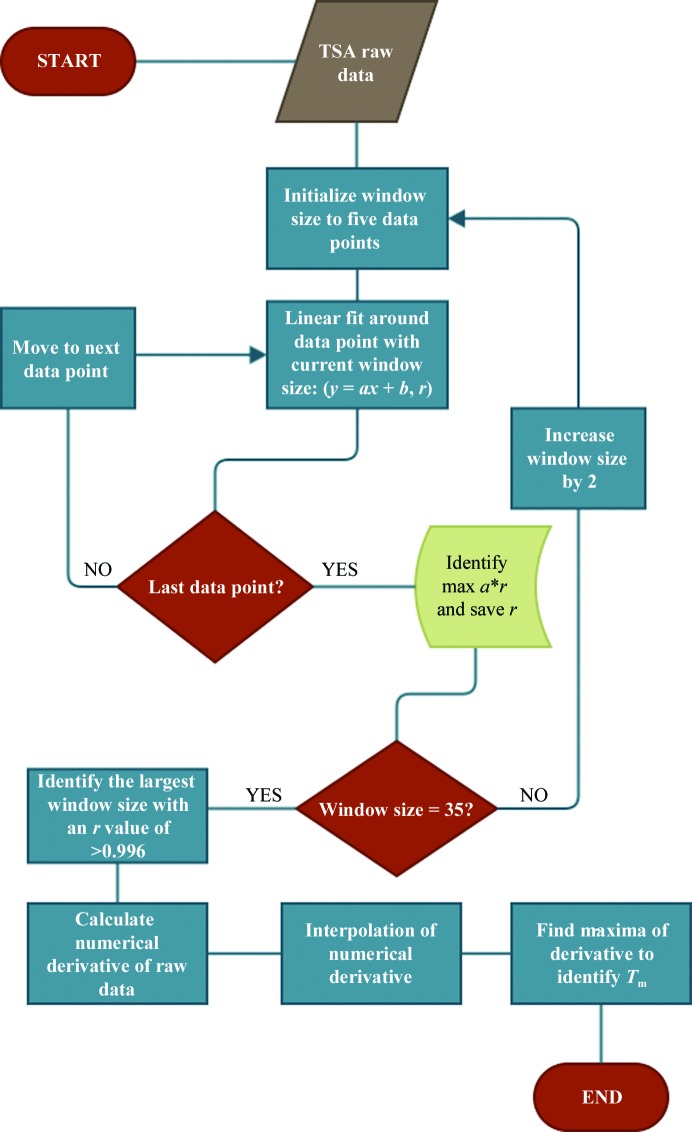
Flow diagram of *NAMI*, a program to analyse thermal shift assay data. Raw data are read in and analysed using a sliding window technique, where a window of increasing number of data points (5–35) is fitted by linear regression, resulting in a slope value (*a*) and a correlation coefficient (*r*) for each window. The optimal window size has been empirically found to be the one with the largest window size and *r* > 0.996.

**Figure 3:**
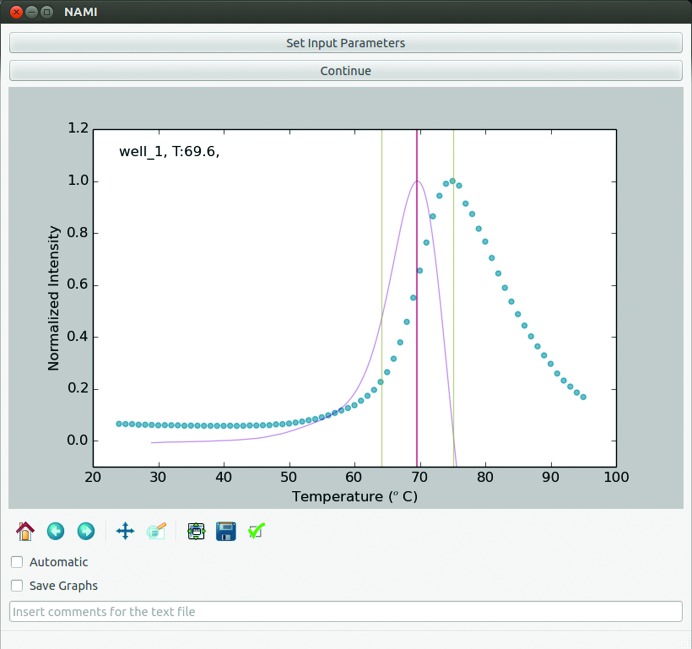
Main graphical user interface for TSA data analysis. The raw data are indicated as blue circles and the interpolated rate of change is shown in purple. The window used for determining *T*_h_ is shown as green vertical lines with *T*_h_ indicated as a red line. Options for automatic running and saving of all curves are indicated.

In order to enable fast and automatic data analysis, a program, *NAMI*, was written in Python. Given the heterogeneity of the data, a purely analytical approach by differentiation of the raw data or a fitted function proved to be suboptimal. Therefore, a numerical approach was chosen to identify *T*_h_. The algorithm is summarized in the flowchart shown in Fig. 2 ▶. First, the fluorescence data and optionally the screen compositions are read as two comma-separated value (csv) text files. The denaturation window is then determined by applying a sliding window over the data set (fluorescence and temperature). The data in each window are fitted by linear regression, resulting in a slope value (*a*) and a correlation coefficient (*r*). This procedure is automatically repeated with increasing window range. The optimal window range has been empirically determined from test measurements from a wide range of proteins to have the steepest slope with a correlation coefficient of *r* = 0.996. This window range is subsequently used to calculate the rate of change for each data point as an approximation for the derivative. In order to calculate a precise *T*_h_, the *UnivariateSpline* function from the *SciPy* package (Oliphant, 2007 ▶) is used to interpolate the experimentally derived data points. Up to two maxima are then reported as possible melting temperatures. Denaturation curves without a clear inflection point are automatically labelled N/S for no signal. The main GUI (shown in Fig. 3 ▶ with one representative melting curve) reads the composition of each experiment from simple text files (csv format) and stores all results in new text files for manual inspection and/or subsequent analysis. These analysis tools will be explained in §[Sec sec4]4.

### 3.2. Dual polarization interferometry   

#### 3.2.1. Cu^2+^ binding to human serum albumin (HSA)   

Human serum albumin (HSA) is a high-molecular-weight endogenous plasma protein of 67 kDa. It is the main component of the blood transport system and reversibly binds a variety of endogenous (vitamins and lipids) and exogenous (drugs and toxins) molecules, distributing them to the target organs (Curry, 2009 ▶). HSA is also known to bind metal cations, which play crucial roles in human growth, development, cell division and synthesis of proteins and DNA. It is essential in the transport and metabolism of competitively bound Cu^2+^ ions in particular (Bal *et al.*, 2013 ▶). Human serum albumin in 20 m*M* Tris, 500 m*M* NaCl, 25 m*M* sodium sulfate pH 5.7 was physisorbed onto an amine chip at a concentration of 4 mg ml^−1^. The running buffer was the same but contained only 150 m*M* NaCl. After immobilizing the protein on the surface, the chip was rinsed in running buffer, followed by a series of blank and CuCl_2_ injections at various concentrations.

## 4. Results and discussions   

### 4.1. Thermal shift assay of glucose isomerase   

**Figure 4:**
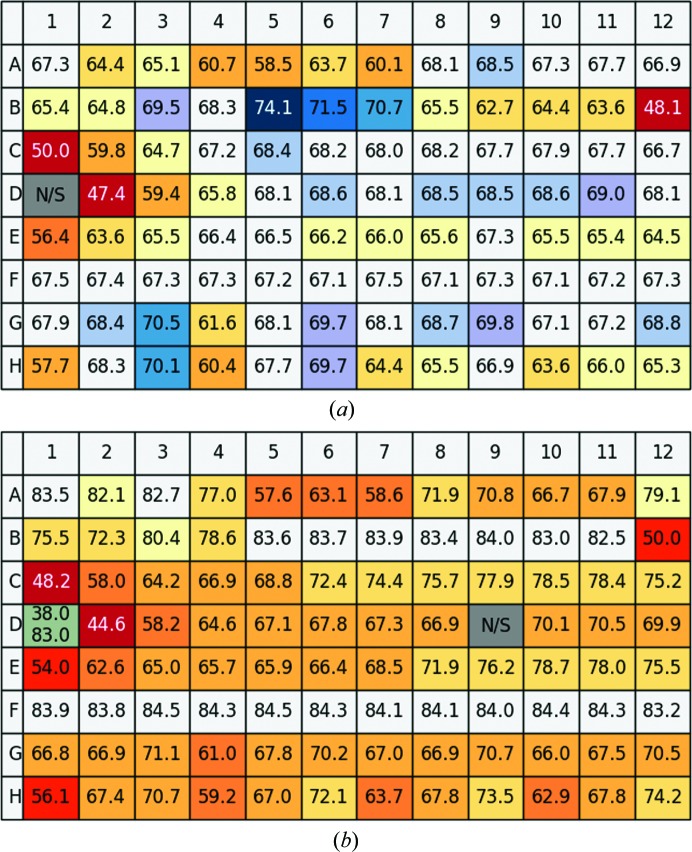
(*a*) Results of a Thermofluor assay with glucose isomerase using the pH screen, showing the melting temperature for each of the 96 wells. The colour-coding scheme indicates increases in melting temperature by a colour change from light blue to dark blue (largest increase) and decreases by colours from yellow to red (largest decrease). Green signifies either two stages in the melting process or heterogeneous data. (*b*) Results of a Thermofluor assay with glucose isomerase using the pH screen with 2.5 m*M* MgCl_2_ added. Note that in this screen row F was used as a control with only water. Position D1 (pH 4) resulted in double peaks and was automatically marked as grey = unreliable. Position D9 contained a foreign particle and hence gave an unreliable result. N/S stands for no signal which could be owing to a lack of sample or to denatured protein.

**Figure 5:**
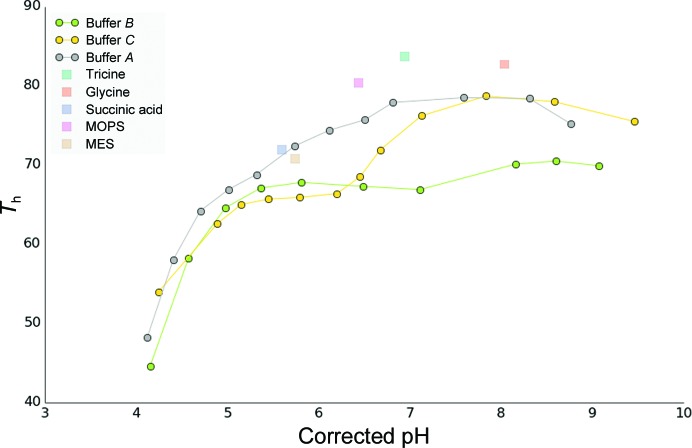
Analysis of the pH screen based on the results in Fig. 4 ▶(*b*). The graph shows the melting temperature *T*_h_ as a function of pH. The pH value is temperature-corrected by Δp*K*_a_ per degree.

After analysing the measurements of each individual well in the 96-well plate, all resulting curves can be displayed and stored (representatives for high-background, two-step denaturation and full denaturation are shown in the Supplementary Fig. S1). The melting temperatures of the TSA experiments with glucose isomerase are summarized in Fig. 4 ▶. The user can display the contents of individual wells by moving the cursor over each well position. The results of the first pH screen (no Mg^2+^ present) shown in Fig. 4 ▶(*a*) indicate two areas that increase the thermal stability, in particular positions B5–B7, corresponding to HEPES, Tricine and Tris buffer pH 7.5–8.1. In addition, positions G3, H3, G6, H6 and G9 corresponding to buffers at pH 9 show a clear increase. The effect of Mg^2+^ was then evaluated by repeating the same screen under identical conditions except for the addition of 2.5 m*M* MgCl_2_ (Fig. 4 ▶ *b*). It is apparent that the effects of buffer and Mg ^2+^ are not simply additive: whereas Mg^2+^ in general increases thermal stability, most of the buffers in combination with Mg^2+^ have the opposite effect. A number of conditions are clearly detrimental to protein stability, such as C1, D2 and B12 (bis-tris pH 6.8, buffer *C* pH 4.4 and phosphate buffer pH 12.4). The program offers a number of ways to further analyse the data using simple menu-driven choices, as exemplified in Fig. 5 ▶, in which the pH profiles of different buffers are summarized. It is evident that buffer *A* (shown in grey) results in a higher thermal stability over a larger pH range compared with buffers *A* and *B* .

**Figure 6:**
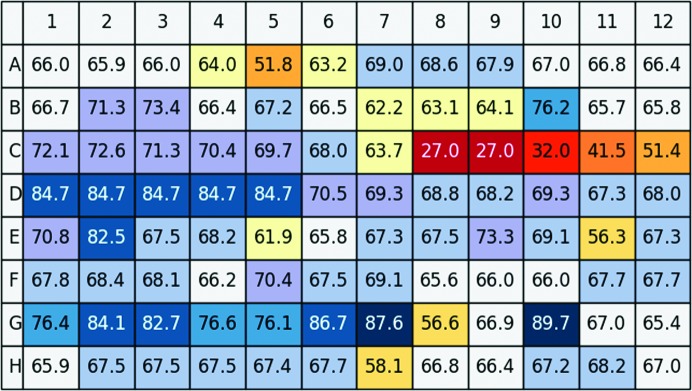
Melting temperatures *T*_h_ for each of the 96 wells of the salt screen obtained with glucose isomerase stripped of Mg^2+^. Increases in *T*_h_ with respect to the three blanks (A1–A3) are indicated in colours from light to dark blue (largest increase) and decreases in yellow to red (maximum decrease).

**Figure 7:**
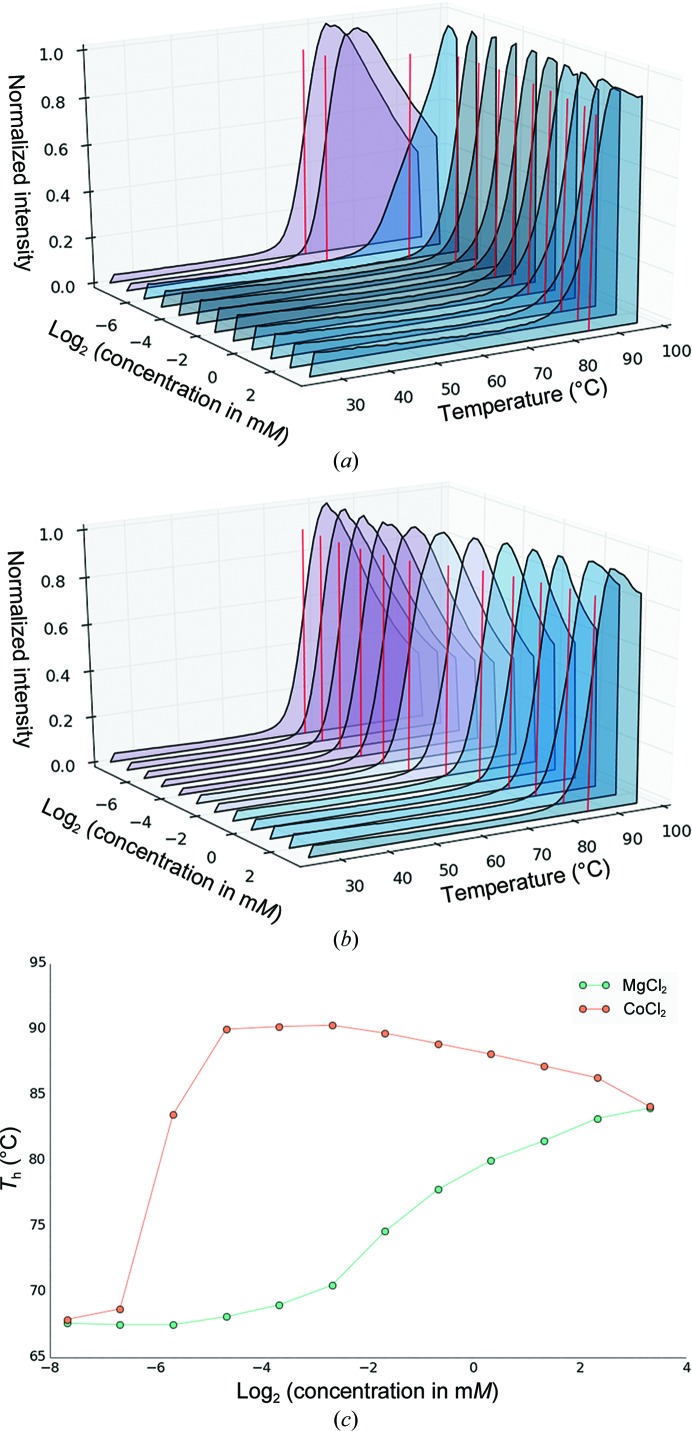
Examples of the analysis part of *NAMI*. (*a*) Waterfall plot of a follow-up screen in which the effect of the serial dilution of divalent metals on glucose isomerase is shown. The starting concentration of CoCl_2_ is 10 m*M*. Purple curves indicate no significant difference from the reference; blue curves indicate a significant shift towards higher *T*_h_. (*b*) Waterfall plot of increasing MgCl_2_ concentration starting at 10 m*M*. (*c*) Melting temperature *T*_h_ as a function of the concentration of CoCl_2_ and MgCl_2_, respectively.

The results for the general salt screen shown in Fig. 6 ▶ confirm that not only Mg^2+^, which is a known cofactor, but a number of other divalent transition metals including Ca^2+^ (position G3 and a known inhibitor of enzyme activity) as well as Co^2+^ (position G7) significantly increase the thermal stability. In this case Na^+^ and NH_4_^+^ appear to have a stabilizing effect; however, these cations have a destabilizing effect when the same screen is repeated with 2.5 m*M* MgCl_2_ in the solution, which may suggest that they interfere with Mg^2+^ binding at high concentrations (data not shown). In order to further investigate the concentration dependency, an additional 96-well screen was produced in which the concentration of a number of divalent transition metals was systematically halved by serial dilution starting from 10 m*M*. The results for Co^2+^ and Mg^2+^ are summarized in Fig. 7 ▶, in which the full fluorescence data of each serial dilution series is shown first as waterfall plots. There is no stabilizing effect at the two lowest concentrations of Co^2+^ (approximately 5 and 10 µ*M*); however, the binding-site concentration is approximately 15 µ*M* (with a protein concentration of 120 µ*M* and eight binding sites; Fig. 7 ▶ *a*). At 20 µ*M* the stabilizing effect of Co^2+^ is evident, with a significant shift in *T*_h_. In addition, the raw data reveal a shoulder at this concentration, which may be owing to partial occupancy of the metal-binding sites.

The stabilizing effect of both divalent metals is clearly visible in Figs. 7 ▶(*b*) and 7 ▶(*c*). However, it is also apparent that increasing the Co^2+^ concentration above the protein saturation decreases the thermal stability.

### 4.2. Dual polarization interferometry   

**Figure 8:**
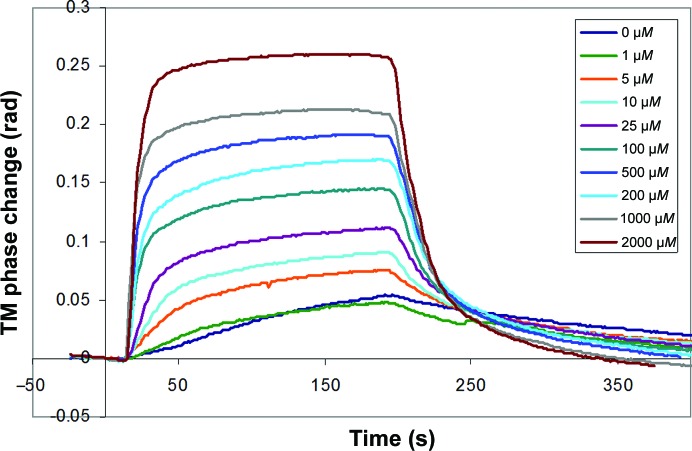
Experimental results of DPI measurements with HSA immobilized on the sensor chip. Raw phase change data in one polarization (transverse magnetic; TM) for a concentration series of Cu^2+^ injections of increasing concentrations.

**Figure 9:**
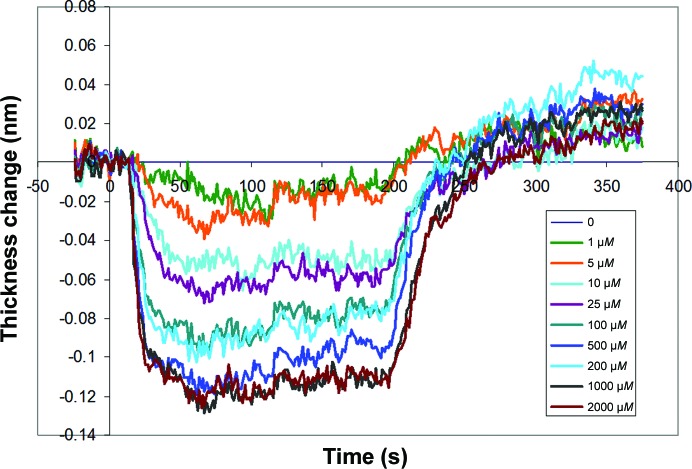
Real-time layer thickness for HSA incubated with Cu^2+^ ions at increasing concentrations.

The DPI experiment was performed to investigate the structural changes associated with the binding of Cu^2+^ ions to a physisorbed layer of human serum albumin (HSA). The protein was adsorbed onto the chip surface and initially formed a layer 4.2 nm thick, which indicates a flat orientation on the surface, as might be expected. This reduced slightly on rinsing to 3.98 nm thick, with a layer protein density of 0.6 g cm^−3^ and a mass per unit area of 2.38 ng mm^−2^ immediately before the first sample injection. The raw data for the copper–HSA interaction for the HSA-coated channel are shown in Fig. 8 ▶. After the blank injection and control channel have been subtracted from the data, the layer thickness, refractive index (RI), protein density (calculated from the changes in refractive index) and mass can be calculated (Cross *et al.*, 2003 ▶). The real-time layer-thickness changes for the raw data are shown in Fig. 9 ▶.

Binding of the Cu^2+^ ions can be seen to compress the HSA structure in a rapid and reversible process, causing a reduction in the layer thickness and a concomitant increase in the layer density (grams per millitre of protein within the HSA layer). HSA binds copper at several different sites, where multiple binding mechanisms cause very different structural changes. This effect can be seen as marked differences in the time or concentration dependence of these plots (Thibault *et al.*, 2006 ▶). Similarly, different ligand interactions can also be differentiated by different conformational changes (Coan *et al.*, 2012 ▶). In this case, thickness and density do not show marked differences; however, a plot of equilibrium thickness *versus* density as a function of copper ion concentration shows a change of slope at ∼10 µ*M* (data not shown), which would indicate that the higher affinity site produces a stronger conformational change.

**Figure 10:**
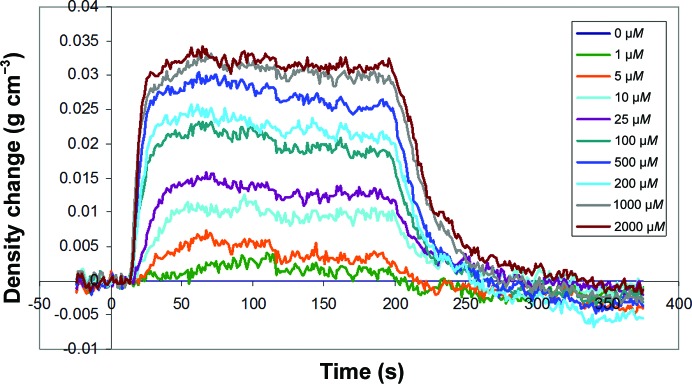
Layer-density change in response to a series of Cu^2+^ injections, showing the concentration-dependent densification of HSA upon Cu^2+^ binding.

**Figure 11:**
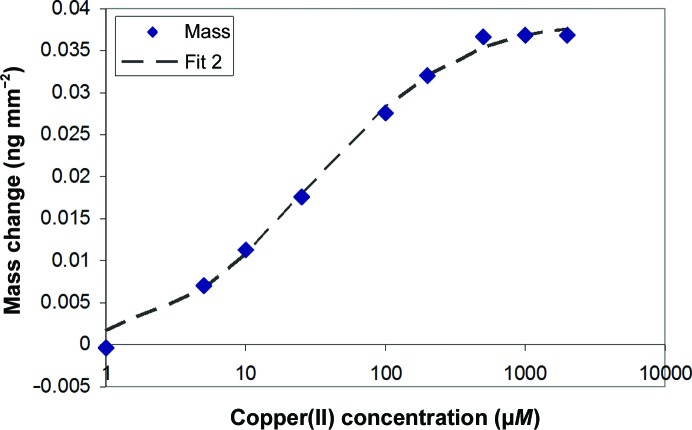
Mass change *versus* copper ion concentration from the experiments shown in Figs. 8 ▶ and 9 ▶. The dashed line corresponds to an affinity fit of two binding sites with copper affinities of 15 and 99 µ*M*.

Affinity constants can be derived from the saturated equilibrium values usually from the phase or mass (Fig. 10 ▶). Here, a single-site affinity model is calculated at 27 µ*M*, which lies within the literature range of 0.23–110 µ*M* (Sokołowska *et al.*, 2010 ▶); however, a double-site affinity curve produces a better fit, with affinity constants of 15 and 99 µ*M* (Fig. 11 ▶).

## 5. Conclusions   

The two methods presented here represent two ends of the wide spectrum of biophysical methods for the characterization of biomolecular interactions. Thermal shift assays can be easily established and performed using standard equipment that is present in almost every molecular-biology or macromolecular laboratory. Examining dose–response relationships, TSA rarely provide a dissociation constant at physiological temperatures but allow the experimenter to distinguish between effects owing to specific binding (such as Co^2+^ binding to glucose isomerase) and nonspecific effects (for example the denaturing of glucose isomerase by increasing concentrations of guanidinium hydrochloride). They do, however, provide a good initial estimate of binding constants, which can facilitate the experimental setup for orthogonal approaches and is one of the few methods that is sensitive enough to examine binding affinities in the millimolar range.

TSA have been widely used to aid crystallization experiments and a number of screens have been described (Nettleship *et al.*, 2008 ▶; Reinhard *et al.*, 2013 ▶). The pH and salt screens presented here are currently being further optimized, as some of the initial conditions showed precipitation when stored at 4°C. One logical extension of the salt screen is the incorporation of heavy-atom compounds such as lanthanides to aid in phasing. TSA experiments are straightforward to adapt to a given set of conditions for a particular protein or problem, and they can easily be used to investigate specific protein–ligand inter­actions. Data can easily be analysed using a range of standard commercial software. Our program *NAMI*, which provides a user-friendly graphical interface coupled with automatic analysis and a wide range of graphical representations, is freely available from Github (http://github.com/grofte/NAMI) under the three-clause BSD open-source licence.

Dual polarization interferometry, which shares many similarities with surface plasmon resonance, offers the advantage that binding events of one partner immobilized on a chip surface can be directly related in real time to alterations in layer thickness and refractive index, as well as mass. This means that in principle there is no *a priori* requirement for a net mass change to elucidate a binding or structural/conformational response: an aspect that has been utilized for studying pH-induced changes in biopolymer (Westwood *et al.*, 2013 ▶) and protein (Sheu *et al.*, 2010 ▶) structures. Hence, DPI not only represents an alternative method to determine binding constants, but can also be used to provide information on molecular orientation and structural changes occurring during binding events. DPI can thus be used to identify and classify ligands with different modes of action leading to different structural changes upon binding.

## Supplementary Material

Supporting Information.. DOI: 10.1107/S1399004714016617/ba5217sup1.pdf

## Figures and Tables

**Table:** Final concentrations of the screen when diluted 50% by addition of protein in buffer. Position A1 is used as the reference value. Buffers *A*, *B* and *C* are composite/universal buffers (Newman, 2004 ▶) used at a concentration of 0.2*M*. The composition of each buffer is given in [Sec sec2]2.

	1	2	3	4	5	6
A	H_2_O	0.1*M* boric acid pH 9.2	0.1*M* glycine pH 9.6	4.5*M* urea	0.1*M* succinic acid pH 4.2	0.1*M* acetic acid pH 4.8
B	0.1 *M* bis-tris propane pH 6.8	0.1 *M* imidazole pH 6.95	0.1 *M* MOPS pH 7.1	0.1 *M* phosphate pH 7.2	0.1 *M* HEPES pH 7.5	0.1*M* tricine pH 8.0
C	Buffer *A* pH 4.0	Buffer *A* pH 4.4	Buffer *A* pH 4.7	Buffer *A* pH 5.0	Buffer *A* pH 5.4	Buffer *A* pH 5.6
D	Buffer *B* pH 4.0	Buffer *B* pH 4.5	Buffer *B* pH 4.9	Buffer *B* pH 5.4	Buffer *B* pH 5.9	Buffer *B* pH 6.1
E	Buffer *C* pH 4.0	Buffer *C* pH 5.0	Buffer *C* pH 5.6	Buffer *C* pH 5.8	Buffer *C* pH 6.3	Buffer *C* pH 6.8
F	H_2_O	H_2_O	H_2_O	H_2_O	H_2_O	H_2_O
G	Buffer *B* pH 5, 0.8*M* malonate	Buffer *B* pH 7, 0.8*M* malonate	Buffer *B* pH 9, 0.8*M* malonate	Buffer *B* pH 5, 0.4*M* malonate	Buffer *B* pH 7, 0.4*M* malonate	Buffer *B* pH 9, 0.4*M* malonate
H	Buffer *C* pH 5, 0.8*M* malonate	Buffer *C* pH 7, 0.8*M* malonate	Buffer *C* pH 9, 0.8*M* malonate	Buffer *C* pH 5, 0.4*M* malonate	Buffer *C* pH 7, 0.4*M* malonate	Buffer *C* pH 9, 0.4*M* malonate

**Table:** 

	7	8	9	10	11	12
A	0.1*M* citric acid pH 4.8	0.1*M* succinic acid pH 5.6	0.1*M* MES pH 6.3	0.1*M* citric acid pH 6.4	0.1*M* ADA pH 6.6	0.1*M* bis-tris pH 6.75
B	0.1 *M* TrisHCl pH 8.1	0.1 *M* bicine pH 8.5	0.1 *M* bis-tris propane pH 9.0	0.1 *M* CHES pH 9.5	0.1 *M* CAPS pH 10.3	0.1 *M* phosphate pH 12.3
C	Buffer *A* pH 6.0	Buffer *A* pH 6.4	Buffer *A* pH 6.9	Buffer *A* pH 7.6	Buffer *A* pH 9.2	Buffer *A* pH 10.0
D	Buffer *B* pH 6.6	Buffer *B* pH 7.4	Buffer *B* pH 7.5	Buffer *B* pH 8.3	Buffer *B* pH 9.1	Buffer *B* pH 10.0
E	Buffer *C* pH 7.4	Buffer *C* pH 7.8	Buffer *C* pH 8.0	Buffer *C* pH 8.5	Buffer *C* pH 9.2	Buffer *C* pH 10.0
F	H_2_O	H_2_O	H_2_O	H_2_O	H_2_O	H_2_O
G	Buffer *B* pH 5, 0.8*M* NaCl	Buffer *B* pH 7, 0.8*M* NaCl	Buffer *B* pH 9, 0.8*M* NaCl	Buffer *B* pH 5, 0.4*M* NaCl	Buffer *B* pH 7, 0.4*M* NaCl	Buffer *B* pH 9, 0.4*M* NaCl
H	Buffer *C* pH 5, 0.8*M* NaCl	Buffer *C* pH 7, 0.8*M* NaCl	Buffer *C* pH 9, 0.8*M* NaCl	Buffer *C* pH 5, 0.4*M* NaCl	Buffer *C* pH 7, 0.4*M* NaCl	Buffer *C* pH 9, 0.4*M* NaCl

**Table:** Final concentrations of the salt screen when diluted 50% by addition of protein in buffer. AS, ammonium sulfate; TMAO, trimethylamine *N*-oxide; TMG, trimethyl glycine.

	1	2	3	4	5	6
A	H_2_O	H_2_O	H_2_O	5m*M* SDS	4.5*M* urea	0.5*M* urea
B	1.5*M* NaCl	1.0*M* NaCl	0.8*M* NaCl	0.6*M* NaCl	0.4*M* NaCl	0.2*M* NaCl
C	1.5*M* Mal	1.0*M* Mal	0.8*M* Mal	0.6*M* Mal	0.4*M* Mal	0.2*M* Mal
D	1.0*M* MgSO_4_	0.8*M* MgSO_4_	0.6*M* MgSO_4_	0.4*M* MgSO_4_	0.2*M* MgSO_4_	1.0*M* Na_2_SO_4_
E	0.5*M* LiCl	0.2*M* LiCl	0.5*M* RbCl	0.2*M* RbCl	0.5*M* CsCl	0.2*M* CsCl
F	0.5*M* sodium formate	0.2*M* sodium formate	0.5*M* sodium malate	0.2*M* sodium malate	0.5*M* Na_2_NO_3_	0.2*M* Na_2_NO_3_
G	0.4*M* MgCl_2_	5m*M* MgCl_2_	5m*M* CaCl_2_	5m*M* SrCl_2_	1m*M* ZnCl_2_	1m*M* NiCl_2_
H	5m*M* Na_2_HPO_4_	5m*M* Na_3_VO_4_	5m*M* Na_2_WO_4_	5m*M* Na_2_MoO_4_	20% glycine	10% glycine

**Table:** 

	7	8	9	10	11	12
A	1.5*M* AS	1.0*M* AS	0.8*M* AS	0.6*M* AS	0.4*M* AS	0.2*M* AS
B	1.5*M* (NH_4_)Cl	1.0*M* (NH_4_)Cl	0.8*M* (NH_4_)Cl	0.6*M* (NH_4_)Cl	0.4*M* (NH_4_)Cl	0.2*M* (NH_4_)Cl
C	5m*M* GuHCl	1.0*M* GuHCl	0.8*M* GuHCl	0.6*M* GuHCl	0.4*M* GuHCl	0.2*M* GuHCl
D	0.8*M* Na_2_SO_4_	0.6*M* Na_2_SO_4_	0.4*M* Na_2_SO_4_	0.2*M* Na_2_SO_4_	0.5*M* KCl	0.2*M* KCl
E	0.4*M* NaF	0.1*M* NaF	0.4*M* NaBr	0.1*M* NaBr	0.4*M* NaI	0.1*M* NaI
F	0.5*M* sodium citrate	0.2*M* sodium citrate	0.5*M* sodium lactate	0.2*M* sodium lactate	0.5*M* sodium acetate	0.2*M* sodium acetate
G	1m*M* CoCl_2_	1m*M* CuSO_4_	1m*M* MgCl_2_, CaCl_2_ ZnCl_2_	5m*M* MnCl_2_	5m*M* EDTA pH 8	5m*M* EGTA pH 8.9
H	1*M* arginine pH 7	1*M* TMAO	0.5*M* TMAO	0.5*M* trehalose	1*M* TMG	0.5*M* TMG
