# Supplementary material for: Protein–ligand interactions investigated by thermal shift assays (TSA) and dual polarization interferometry (DPI)
Source: Acta Crystallogr D Biol Crystallogr. 2015 Jan 1;71(Pt 1):36–44. doi: 10.1107/S1399004714016617 (PMC4304684; doi:10.1107/S1399004714016617)
Supplement: Supplementary file 1 [file d-71-00036-sup1.pdf]

BIOLOGICAL
CRYSTALLOGRAPHY

Volume 71 (2015)

Supporting information for article:

Protein–ligand interactions investigated by thermal shift assays (TSA) and dual polarization interferometry (DPI)

Morten K. Grøftehauge, Nelly R. Hajizadeh, Marcus J. Swann and Ehmke Pohl

Figure 1 Main Graphical User Interface for the TSA data analysis. The raw data is indicated as blue circles, the interpolated rate of change is given in purple. The window used for determining T_h is shown on green vertical lines with T_h indicated as a red line. (a) high background (b) two-step process showing the two maxima (c) denatured protein.

(a)

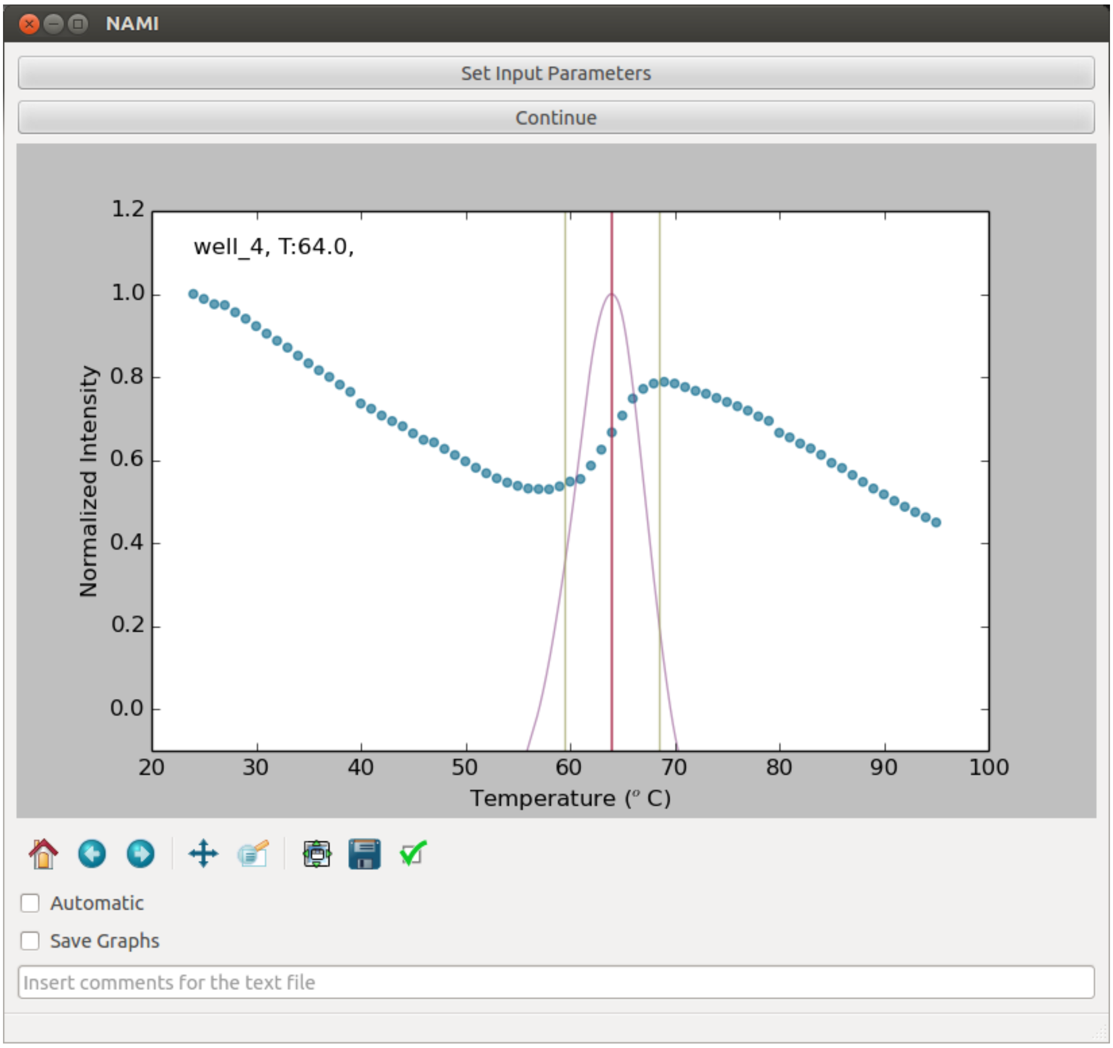

(b)

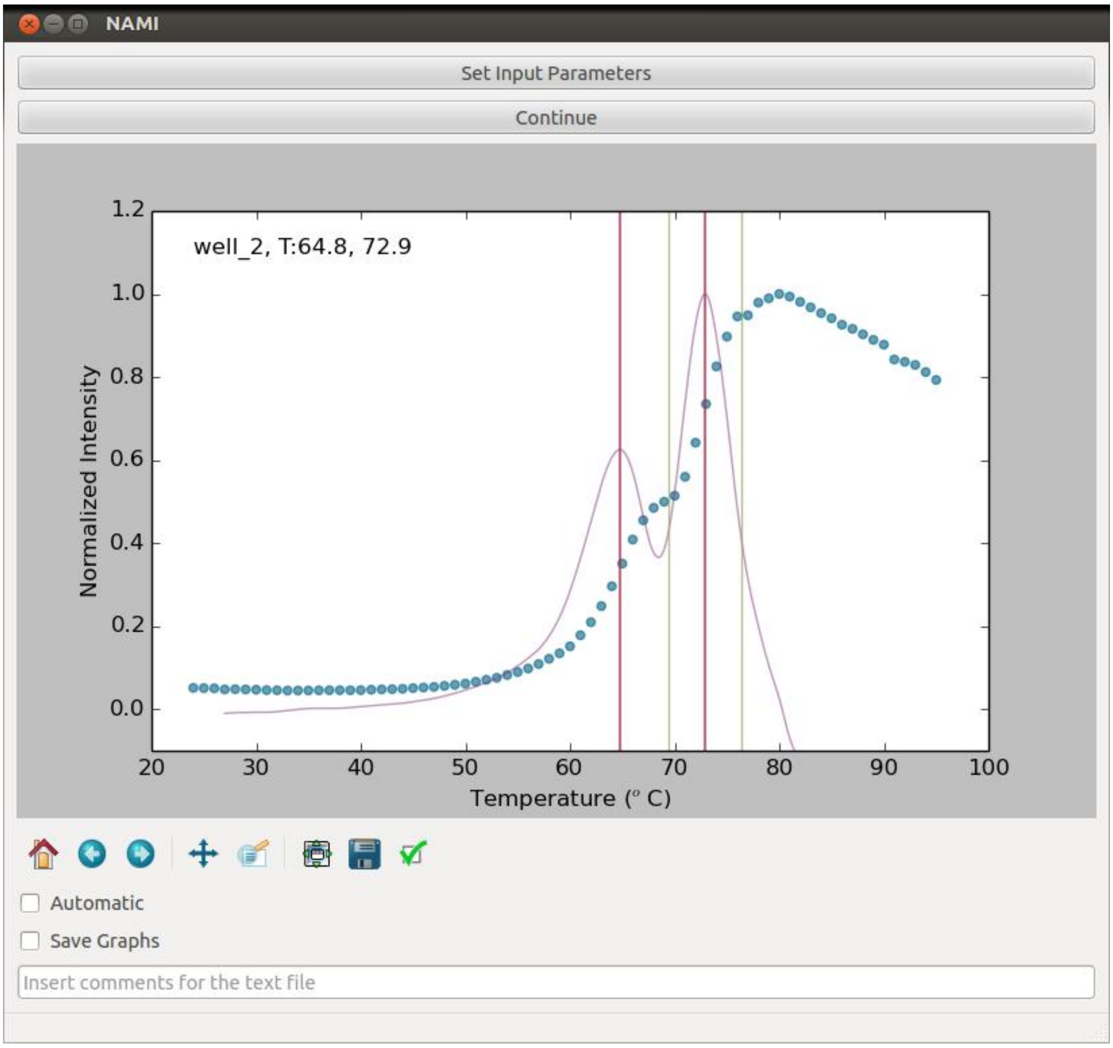
NAMI
Set Input Parameters
Continue
well_2, T:64.8, 72.9
Normalized Intensity
Temperature (° C)
Automatic
Save Graphs
Insert comments for the text file

(c)

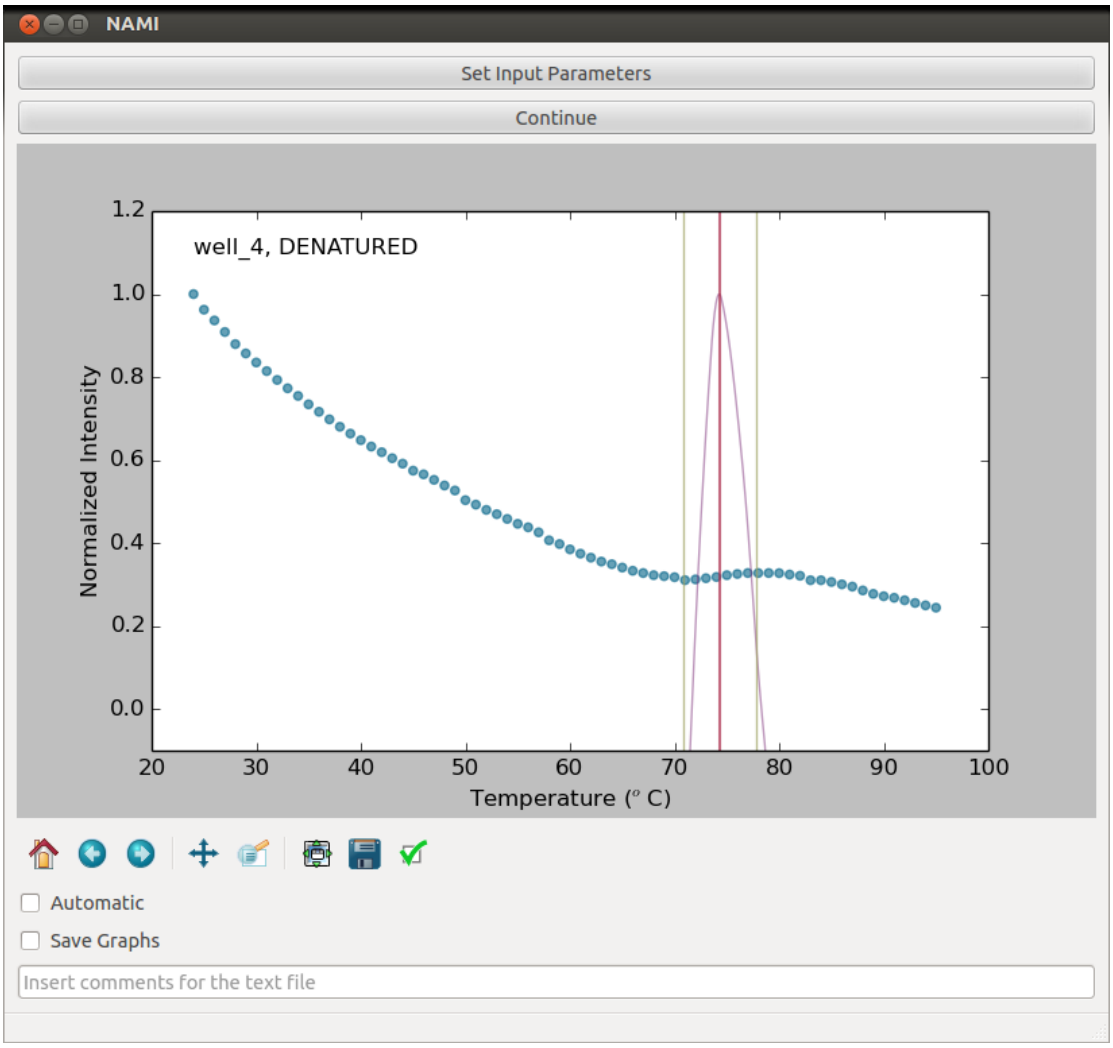
NAMI
Set Input Parameters
Continue
well_4, DENATURED
Normalized Intensity
1.2
1.0
0.8
0.6
0.4
0.2
0.0
20
30
40
50
60
70
80
90
100
Temperature (° C)
Automatic
Save Graphs
Insert comments for the text file
